# Undirected head movements of listeners with asymmetrical hearing impairment during a speech-in-noise task

**DOI:** 10.1016/j.heares.2011.10.009

**Published:** 2012-01

**Authors:** W. Owen Brimijoin, David McShefferty, Michael A. Akeroyd

**Affiliations:** MRC Institute of Hearing Research (Scottish Section), Glasgow Royal Infirmary, 16 Alexandra Parade, Glasgow G31 2ER, UK

**Keywords:** HRTF, head related transfer function, ILD, interaural level difference, ITD, interaural time difference, SNR, signal-to-noise ratio, SPL, sound pressure level

## Abstract

It has long been understood that the level of a sound at the ear is dependent on head orientation, but the way in which listeners move their heads during listening has remained largely unstudied. Given the task of understanding a speech signal in the presence of a simultaneous noise, listeners could potentially use head orientation to either maximize the level of the signal in their better ear, or to maximize the signal-to-noise ratio in their better ear. To establish what head orientation strategy listeners use in a speech comprehension task, we used an infrared motion-tracking system to measure the head movements of 36 listeners with large (>16 dB) differences in hearing threshold between their left and right ears. We engaged listeners in a difficult task of understanding sentences presented at the same time as a spatially separated background noise. We found that they tended to orient their heads so as to maximize the level of the target sentence in their better ear, irrespective of the position of the background noise. This is not ideal orientation behavior from the perspective of maximizing the signal-to-noise ratio (SNR) at the ear, but is a simple, easily implemented strategy that is often effective in an environment where the spatial position of multiple noise sources may be difficult or impossible to determine.

## 1 Introduction

The level of a sound at the ear depends on a number of factors including the distance, direction, and power of the source, as well as the shape and size of the head and outer ears (Blauert, 1983). Of these factors, listeners typically have control over only two of them: direction and distance. Thus if listeners wish to increase the level of a sound, they can either move towards the sound source, turn their heads, or both. In some situations it may not be feasible to move closer to a sound source, leaving head turns as the primary means by which a listener might increase the audibility of a signal. Head movements, on the other hand, change the level at the ear of *any* sound, including distracting noises. In the typical situation of listening to speech in noise, these movements could be used to increase the audibility of the signal in two general ways: listeners could either turn their heads so as to increase the level of the signal in their better ear, or they could turn so as to increase the *difference* in level between the signal and the noise. Of the two, the latter strategy would generally be the better, as the resulting gain in signal-to-noise ratio (SNR) would always be expected to lead directly to an increase in the intelligibility of the signal (Lavandier and Culling, 2010; Rhebergen and Versfeld, 2005), whereas movements to maximize signal level per se can result in a concomitant increase in noise level and therefore no guarantee of a change in intelligibility.

Head movements have historically been studied for their contribution to resolving the location of an auditory signal (Wightman and Kistler, 1999; Thurlow et al., 1967; Young, 1931) and have generally been studied using paradigms that explicitly direct listeners to move. In a previous experiment we demonstrated that adult listeners with hearing impairment orient reliably to sound when given explicit instructions to do so, although hearing impairment is associated with a large increase in the complexity and duration of the orienting responses (Brimijoin et al., 2010). This and other directed search tasks capture some aspects of natural head orientation, but listeners are still asked to move (Simpson et al., 2005).

Undirected movements have been studied less frequently. It has been shown that infants reflexively turn their heads in response to sound (Muir and Field, 1979) and turn to face their caregivers in one-on-one situations (Ching et al., 2009). Adult listeners turn their heads in response to sound when instructed to do so (Brimijoin et al., 2010; Fuller, 1992, 1996), but do not tend to make reflexive orienting movements simply because they hear a sound, although this depends to a large degree on the novelty of the sound (Sokolov, 1963). Undirected head movements have also been studied in social contexts such as turn-taking behavior in conversations. Although there are substantial sex differences, it is clear listeners tend to point their heads towards a talker and that the patterns of these orientations provide a structure to the interaction (Kendon, 1967).

In order to systematically evaluate the use of head orientation as a listening strategy for speech comprehension in an undirected paradigm, we designed a speech task with a spatially-separated signal and noise in which both the signal level and SNR would vary predictably as a function of a listener’s head orientation. Listeners were free to move at any point in the experiment and were not told that they were being tracked. We attempted to create conditions in which a listener’s comprehension might benefit from an optimal head position, encouraging them to move without instruction, so avoiding explicitly directing listeners to orient towards a particular sound source as we did previously (Brimijoin et al., 2010). To this end we used an adaptive speech-in-noise task, varying the signal level. Note that it was not our intent to accurately measure speech reception threshold, rather this procedure served as a simple means by which the task could be made difficult in order to encourage listeners to turn their heads. Indeed, at a high SNR, listeners should able to perform the task using any head orientation; we therefore used the adaptive track to find SNRs at which the listener’s comprehension would benefit from adopting a particular head orientation. To evaluate the strategies used by listeners, we used acoustical measurements made with artificial mannequins to estimate signal level and SNR as a function of head angle. We recruited listeners with large (>16 dB) differences between their right and left ears, reasoning that natural head turning behavior would be strongest for such listeners, especially if given a task to be performed under a poor SNR. This restriction means that this study only considers monaural cues at the better ear.

**Fig. 1:**
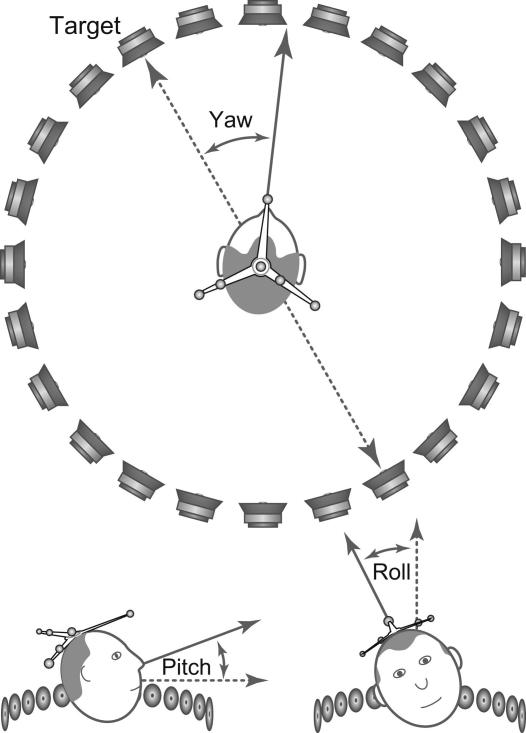
A schematic of the loudspeaker ring and infrared markers. Listeners were seated at the center of a 1.9 m diameter ring of 24 loudspeakers arranged in 15° intervals. Listeners wore a crown to which were attached an array of retroreflective markers. The 3D positions of these markers were used to establish the yaw pitch and roll and the listener’s head. Yaw is reported relative to the target loudspeaker, pitch relative to the plane of the loudspeaker ring, and roll relative to the vertical axis.

Using an infrared motion-tracking system, we measured each listener’s head orientation and position. All orientation data in this study are reported in the form of degrees of yaw, pitch, and roll, specified relative to the target loudspeaker (see Fig. 1). Positive yaw values indicate a head turn in the horizontal plane to the right of the target and negative values indicate a head turn to the left of the target; positive pitch values indicate that a listener is looking upwards, negative that they are looking downwards; positive roll values indicate a tilt of the head to the right, negative to the left.

## 2 Materials and methods

### 2.1 Participants

Listeners were recruited from the local audiological clinic population based on the magnitude of their audiometric asymmetry. We collected data on 20 left-ear listeners and 16 right-ear listeners. The mean age of the left-ear listeners was 61 years; their mean hearing threshold at 500, 1000, 2000, and 4000 Hz was 59 dB in their right ears and 32 dB in their left ears, a 27 dB difference. The mean age of the right-ear listeners was 63 years; their mean threshold was 34 dB in their right ears and 62 dB in their left ears, a 28 dB difference. Listeners were free to move their heads, but they were not informed where to orient their heads, or indeed that the purpose of the experiment was to measure orientation. Listeners with hearing aids were asked to remove them prior to testing. The experiment was conducted in accordance with procedures approved by the West of Scotland Research Ethics Service.

### 2.2 Stimuli

The stimuli were short sentences, about 2–3 s in duration, drawn from the Adaptive Sentence List corpus (MacLeod and Summerfield, 1987). They were presented from one of 24 loudspeakers arranged in a 1.9 m diameter ring, separated by 15° (see Fig. 1). Target sentences were presented in blocks from 105°, −45°, 60°, −30°, and −90°. Speech-shaped noise (ICRA (Dreschler et al., 2001)) was presented concurrently with the target sentences at 70 dB sound pressure level (SPL) at five separations of ±180°, −90°, −30°, +30°, or +90° from the target. All conditions were repeated five times. In five instances for listeners with higher hearing thresholds, the signal and noise were started at 88 dB SPL. Despite this increase in level, for two listeners the signal level during some portions of an adaptive track dropped to within 10 dB of their average pure tone threshold (measured at 500, 1000, 2000, and 4000 Hz). These data were discarded from the final analysis, so giving data on 19 left ear and 15 right-ear listeners.

### 2.3 Experimental protocol

**Fig. 2:**
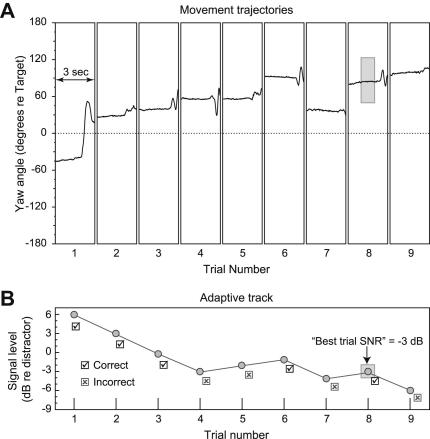
Yaw angle during an adaptive track. The top panel (A) plots an illustrative listener’s head yaw as a function of time for a set of nine trials. Negative values indicate orientation to the left and positive values indicate orientation to the right of the target loudspeaker. The bottom panel (B) shows the level of the target sentence varying over the course of the adaptive track. When the listener correctly repeated the target sentence (black checkmarks), the target was made 3 dB less intense on the subsequent trial. When the listener failed to repeat the target (gray × marks) the target was made 1 dB more intense on the subsequent trial. The trial with the lowest level accurately repeated sentence was selected for further analysis (indicated by the gray boxes in 2A and B), the SNR from this trial was termed the “best trial SNR.”

Listeners were seated in the center of the loudspeaker ring and instructed to repeat as much as they could of the target sentences. They were told that the chair on which they were sitting could rotate and they should feel free to turn if they liked. A wireless lapel microphone allowed the experimenters to determine the accuracy of the listener’s response and adjust the target level accordingly. For each correct answer the level of the target sentence was reduced by 3 dB; for each incorrect answer the level was increased by 1 dB (see Fig. 2B). This adaptive track was terminated after four reversals. Note that this technique and the small number of reversals could not result in precise measurements of speech reception threshold; as noted earlier the adaptive procedure was a convenient means by which the auditory discrimination task could be made difficult, thereby encouraging listeners to orient their heads relative to the target. Threshold is typically computed as a mean of several points along the adaptive track, but because we required discrete level values to compare with discrete head angles, the reported values for each track are the lowest SNR at which the listener correctly repeated the sentence (the gray box in Fig. 2B), henceforth referred to as “Best Trial SNR.” For the purposes of group averages these values for each listener were normalized to his or her mean SNR. Listeners were given a 5 min training session that was identical to the experimental blocks, but with the experimenter present so as to allow the listeners to ask questions if they were unclear on the task.

### 2.4 Motion tracking

Motion tracking was performed using a commercial infrared camera system (Vicon MX3+) using the same methods that we have described previously (Brimijoin et al., 2010). Six cameras were placed above the listener, behind and ahead, and were pointed towards the listener. The system tracked 9-mm diameter reflective spheres; these markers were placed on four of the loudspeakers (at eccentricities of 0°, −90°, +45°, and +90°), a head-mounted “crown” worn by the listeners, and a wand. The motion-tracking system returned three-dimensional Cartesian coordinates of all markers on all objects at a sample rate of 100 Hz. The loudspeaker markers provided reference directions and the wand was used to determine the location of the ears and nose relative to the crown. Coordinate translation and rotation allowed us to determine the true orientation of the listener relative to the loudspeaker ring to within 0.2° regardless of the placement of the crown. A square wave pulse sent by the stimulus PC to the motion-tracking hardware synchronized the orientation data to the stimulus presentation. All angles in the study are averages drawn from the middle third of the presentation of the stimulus and reported relative to the location of the target loudspeaker, wherever it might have been on a given trial. In a few instances the motion-tracking system failed to capture the listener, thus the total number of data points in our analysis is slightly less than the number of listeners multiplied by the five repeats of each condition.

### 2.5 Signal level measurements and SNR prediction

To measure the signal level that listeners would experience as a function of head angle, we presented speech-shaped noise through each loudspeaker in turn (i.e., in 15° increments) and recorded the level at the left ear of a mannequin head and torso simulator with pinnae installed (B&K). To predict the expected SNR benefit as a function of both head angle and distractor angle, we used Lavandier and Culling’s (2010) binaural speech intelligibility model that was converted to a monaural model to reflect our asymmetrical listeners. The model was modified by removing the computation of binaural masking level difference: SNRs were then computed using head related transfer functions (HRTF) which were weighted frequency channel-wise by speech intelligibility indices (ANSI, 1997) and summed to yield a prediction of overall speech intelligibility. We estimated SNR using a signal at 0° and noise from −180° to +180° in 5° increments. The HRTFs used in the model were those of the MIT KEMAR mannequin (Gardner and Martin, 1995).

### 2.6 Statistical analysis

Being drawn from an angular distribution, our data for left- and right-ear listeners failed the Shapiro–Wilks test of normality in SPSS (*W*_(462)_ 0.97, *p* 0.001; and *W*_(372)_ 0.95, *p* 0.001, respectively). Therefore all statistical analyses were performed using Matlab and a parametric two-way ANOVA designed specifically for circular distributions (Berens, 2009; Harrison and Kanji, 1988).

## 3 Results

Fig. 2 contains example data from a listener with a lower audiometric threshold in her left ear. It plots her head yaw, relative to the target loudspeaker (top panel), and the SNR (bottom panel), for each trial in one track. Fig. 2A shows that the listener started the block of trials oriented approximately 45° to the left of the target and made a large orienting movement in the first trial that ended roughly 45° to the right of the target. On subsequent trials, the listener made smaller movements, always remaining oriented to the right of the target. We selected the trial corresponding to the lowest level accurately reported sentence and measured the mean head yaw during the center third of the presentation of the sentence. This is represented as a gray box in Fig. 2A outlining the analysis window.

### 3.1 Yaw results

**Fig. 3:**
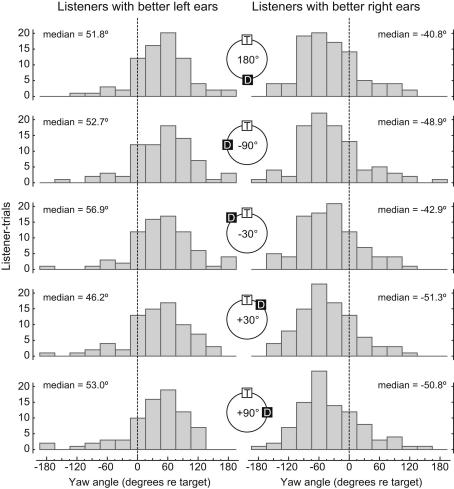
Head yaw for left-ear and right-ear listeners. The top pair of histograms show the results for the ±180° distractor condition, with left-ear listeners on the left, and right-ear listeners on the right. The rows of histograms underneath represent data for the remaining distractor conditions of −90, −30, +30, and +90° respectively.

The distribution of head yaws demonstrated a distinct left-right behavioral bias in our listeners. The top left panel in Fig. 3 plots the distribution of orientation angles (in 30° bins) for listeners with better left ears during the ±180° distractor condition, i.e., when the distractor was diametrically opposite the target. The *y*-axis represents listener-trials (i.e., each listener repeated each condition 5 times). The data shows that these listeners – henceforth termed “left-ear listeners” – tended to orient to the *right* of the target: the median value was +51.8°, the modal value was +60°. The top right panel in Fig. 3 shows the corresponding data for “right-ear listeners.” These data show that these listeners oriented to the *left* of the target: the median was −40.8° and the mode was −60°. The remaining rows of Fig. 3 contain data for the other four distractor conditions of −90°, −30°, +30° and +90°, with left-ear listeners on the left and right-ear listeners on the right. The overall median for left-ear listeners was +51.2° (interquartile range 12.2–79.8°), but for right-ear listeners it was −48.6° (−9.5 to −78.8°). The broad distributions reflect a high degree of variability in listener orientation. We found no systematic change in distributions over the course of the experiment. Using a two-way ANOVA, we found that left versus right-ear listeners differed significantly from each other (*F*_(1,830)_ 396.64, *p* 0.001). No effect of distractor direction was found (*F*_(4,830)_ 0.63, *p* 0.64), however, demonstrating that the distributions of orientation angle were no different for each distractor condition. That is, listeners tended to orient themselves with respect to the target sound irrespective of the position of the distractor sound.

### 3.2 Pitch and roll results

We also recorded head pitch and roll relative to the plane of the loudspeaker ring. A change in pitch rotates the head up and down, but keeps the ears level in the azimuthal plane. All listeners consistently pitched their heads downwards during the task. We found that right-ear listeners had a median pitch of −7.5° and left-ear listeners had a median pitch of −6.2° while listening; this difference was not significant (*F*_(1,830)_ 0.47, *p* 0.76). The lack of difference in pitch between the two groups of listeners is explainable in that audiometric asymmetry differs from left to right ears in the azimuthal plane only, but not in elevation. We also found no effect of distractor direction on pitch (*F*_(4,830)_ 0.58, *p* 0.68).

Both groups of listeners rolled their heads to the right during the task; left-ear listeners rolled their heads +2.5° and right-ear listeners rolled +2.7°. There was no effect of listener type (*F*_(1,830)_ 1.17, *p* 0.28) or distractor angle (*F*_(4,830)_ 0.56, *p* 0.69) on roll. Combined with the typical yaw orientation this means that for right-ear listeners the direction of the target was found to the right and slightly above the plane of the two ears; but for left-ear listeners, on the other hand, the direction of the target was found to the left and slightly below the plane of the two ears.

### 3.3 Signal level and SNR as a function of head orientation

**Fig. 4:**
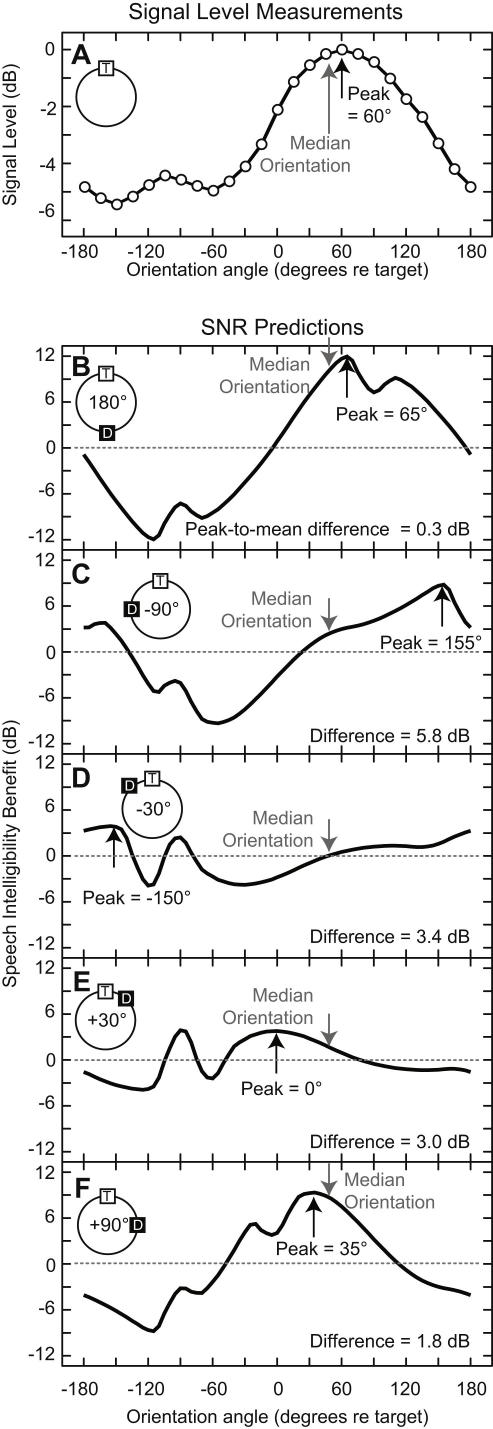
Head yaw and predicted speech intelligibility. A speech intelligibility model predicted the yaw angle at which listeners would receive the greatest benefit (in decibels) in understanding a target sentence paired with a simultaneous noise distractor. For each of the distractor conditions (stacked vertically as in the previous figure) the black upward arrows indicate the peak of these curves. The gray arrows indicate the median yaw behavior of listeners.

Fig. 4A plots the results of our mannequin recordings. *Signal level* varied as a function of head angle by 5.5 dB and was (for left-ear listeners) maximal at +60° head orientation. This angle is approximately the same as the median orientation angle adopted by listeners (gray arrow). We used a modified version of Lavandier and Culling’s (2010) model to predict SNR as a function of head angle. These results are plotted in the remaining panels of Fig. 4B–F. The model, set to left-ear listening, predicted that speech intelligibility would be maximal at head yaws of +65°, +155°, −150°, 0°, and +35° for the distractor angles of ±180°, −90°, −30°, +30°, and +90°, respectively. Thus only in the case of the 180° distractor condition was the best SNR was found within 5° of the angle which gives maximum signal level (Fig. 4B). For all other distractor conditions, the best SNR and maximum signal level were found at different angles (Fig. 4C–F). The median orientation of listeners (gray arrows) was approximately similar to the ideal orientation for only ±180° and +90°. For other distractor angles, however, the typical orientation adopted by listeners was different from the ideal orientation, in some cases by as much as 100°.

### 3.4 Best trial SNR and head angle

**Fig. 5:**
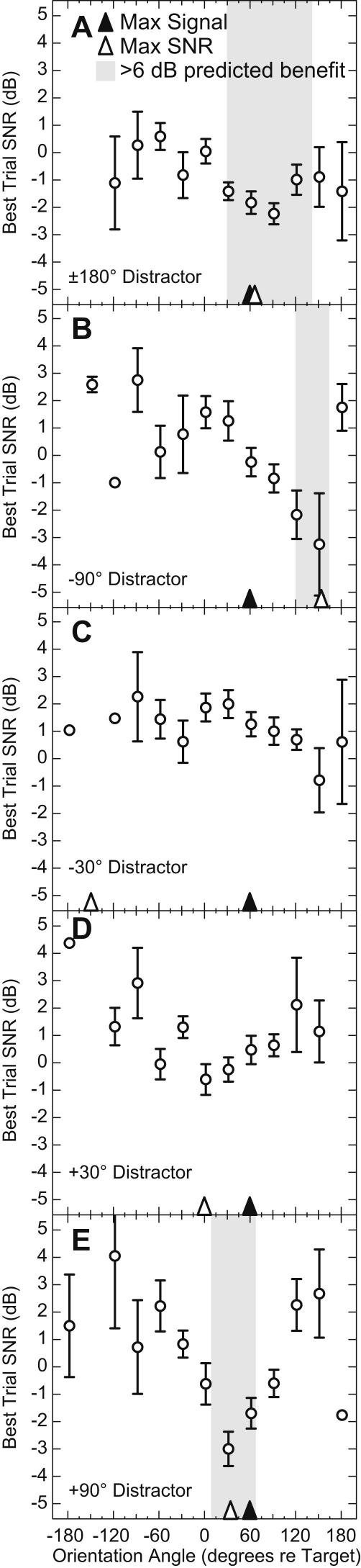
Head yaw and task performance. Performance (best trial SNR) is plotted as function of head yaw for each of the five distractor angles. Error bars are standard error of the mean. The filled arrowheads indicate the orientation angle associated with maximum signal level and the open arrowheads indicate the angle of maximum SNR. The gray bar represents the range of head angles over which the model predicts a listener would receive at least 6 dB of speech intelligibility benefit.

Despite a strategy that appeared to favor maximizing signal level over SNR, listeners did occasionally orient in a direction that offered a good SNR. We found that in these few cases listener performance on the task was better, which is consistent with the well-established relationship between SNR and speech intelligibility. To demonstrate this, Fig. 5A plots listeners’ mean performance (best trial SNR) on the speech reception threshold task as a function of their head yaw, with error bars indicating standard error of the mean. Because the performance of right-ear listeners was indistinguishable (albeit right-to-left mirror-reversed) from that of left-ear listeners, their data was reflected about the 0° point and included in the means with the left-ear listeners; thus all listeners are plotted as if they had better left ears. For the 180° distractor condition, yaw angles to the right of the target tended to be associated with lower best trial SNR. This data reflects the increase in speech intelligibility that is the result of turning the better ear towards the target. The gray bar in the figure represents the range of head angles over which the Lavandier and Culling model predicts a listener would receive at least 6 dB of speech intelligibility benefit. The three lowest best trial SNR averages are all found within this bar. The yaw angle associated with the maximal signal level is indicated with a solid arrow, the orientation angle associated with maximal SNR is indicated with a dashed arrow. Both angles are fairly close to the angle with the lowest best trial SNRs of the listeners.

Fig. 5B plots the data for the −90° condition and shows a more complex dynamic between orientation angle and performance. In this case, orientation angles to the right of the target were not always accompanied by low best trial SNRs. The angle corresponding to maximal signal level was (as before) 60°, but the angle associated with maximum predicted SNR was 155°. Listeners’ best performance corresponded to trials in which they oriented to directions close to those that were predicted to yield the best SNR (note that the two lowest values are found within the gray bar). Listeners did not often orient to such large angles (see Fig. 3), although on the few occasions that they did, they performed far better than when oriented to 60°. The data for the −30° and +30° conditions (Fig. 5C and D, respectively) demonstrated a less clear pattern, possibly reflecting the reduced SNR advantage available with small subtended angles between target and distractor. Note that for these two conditions the model didn’t predict an intelligibility benefit greater than 6 dB (no gray region in either panel), suggesting that with targets and distractors separated by small angles, no head angle can provide a substantial benefit. Unlike the previous distractor angles, no clear advantages in terms of lower best trial SNRs were found associated with maximum SNR. Fig. 5E plots best trial SNRs for the +90° condition. Listeners’ lowest level correctly answered sentences were found at orientation angles closer to the angle associated with maximum predicted SNR than to that associated with maximum level.

## 4 Discussion

### 4.1 Main findings

It is perhaps unsurprising that listeners with strong audiometric asymmetries tend to actively utilize head orientation during a listening task. They have much to gain from such behavior: depending on the angular separation between the target signal and whatever distractors may be present, an optimal head angle can give several decibels of benefit in signal-to-noise ratio at the ears. Listeners do not, however, appear to use SNR to guide their head orientation. Given the clear tendency for listeners to orient to about 60° away from the target irrespective of the position of the distractor signal, it is clear that listeners did not take into account the position of the distractor. Because the head angle for optimal SNR varies with distractor position, listeners could not have been seeking to maximize SNR. As an orientation angle of 60° corresponds to the maximum signal level, we suggest that listeners were instead seeking to maximize signal level. From the perspective of speech intelligibility this is not ideal behavior, but it probably reflects a number of factors that are critical in real-world listening.

### 4.2 Non-acoustic factors in orientation

Listeners in the real world likely use head orientation to balance numerous factors relating to speech understanding and social convention. For some combinations of target and distractor angles, the speech intelligibility model suggests that the ideal behavior – assuming that the goal is to maximize SNR – is to turn away from the target sound. In the case of a listener with a better left ear and a distractor at −90° for example, the listener must turn 155° away from the target in order to maximize the SNR. This orientation, nearly fully facing away from the target, is decidedly inappropriate in a conversational setting. Listeners in a social setting tend to look at the person talking (Kendon, 1967). In fact, gaze (the combination of head and eye angle) has often been cited as a major social factor in the structure of conversations both for listeners and talkers, signalling among other things readiness to talk and the imminent yielding of the floor (Kendon, 1967). Thus in at least some of the target/distractor separations tested, maximizing SNR conflicts directly with normal social behavior.

Maximizing SNR also conflicts with a second possible constraint on real world orienting behavior: being able to see lip movements or related head movements helps speech comprehension substantially (e.g., Sumby and Pollack, 1954; Erber, 1969; Grant, 2001; MacLeod and Summerfield, 1987; Schwartz et al., 2004). To gain a benefit from speech reading, however, one’s head must be turned towards the talker. The human eye tends never to fixate more than ±45° off the midline (Guitton and Volle, 1987) and the accuracy of speech reading decreases rapidly as a function of angular distance from the fovea (Smeele et al., 1998). Thus for only two of the configurations we tested (distractors at +30° and +90°) would the ideal SNR orientation (0° and +35°, respectively) allow any substantial benefit from lip reading. It should be noted, however, that in the present study there was no advantage to visualizing the target loudspeaker as we did not present any visual stimuli. It remains for future work to determine the effect that adding visual information has on orienting behavior.

### 4.3 Methodological caveats

This study was not designed to test performance as a function of head angle, rather it was designed simply to determine what head orientation a listener would naturally use when confronted with a particular signal and noise configuration. An undirected paradigm in which listeners are free to turn as they wish, however, naturally results in a heterogeneous data set; this variability makes meaningful averages of behavior problematic. The SNR values in Fig. 5 represent means of different numbers of listeners ranging from 1 to 33. This is unavoidable given how our experiment was constructed. That said, the data suggest that listeners perform better when they orient to angles that are associated with better SNR. This finding is in accord with literature demonstrating the strong relationship between SNR and speech intelligibility (French and Steinberg, 1947; Hawkins and Stevens, 1950). Sensitive tests of performance as a function of head angle must be conducted in a paradigm in which listeners are directed to orient in specific ways.

Because HRTFs are highly specific from listener to listener it is possible that the KEMAR HRTFs (Gardner and Martin, 1995) we used to predict speech intelligibility may not adequately capture some specific cues that could be used by individual listeners. We are, however, confident that in spite of the individuality of HRTFs, the primary contributors to speech intelligibility as a function of head angle in the horizontal plane are interaural time difference (ITD) and interaural level difference (ILD) cues, which are both adequately modelled by KEMAR. It should also be noted that in addition to their large yaw movements, listeners pitched and rolled their heads by a small amount. Head pitch and roll do have consequences for the acoustics of the HRTF but were not included in the model because the magnitude of the pitch and roll angles we found were smaller than the sampling resolution of the MIT HRTF database in the vertical plane (10°). We argue that such small pitch and roll angles would not radically alter the far larger effects of yaw on ITD and ILD in the azimuthal plane.

Finally, this study only considered monaural contributions of head position on speech intelligibility. This simplification allowed us to take advantage of the large range in predicted SNRs for monaural listeners and was chosen to test whether listeners would be capable of determining and using the best head angle for speech comprehension in noise. Future work should examine the orienting behavior of listeners with symmetrical hearing, both normal and impaired.

### 4.4 Summary

In summary, our data demonstrate that the functional advantage of increased signal-to-noise ratio is disregarded in favor of a behavior that emphasizes signal level. Using head orientation to maximize SNR, however, requires a more complex behavioral strategy than does simply maximizing target level, as it requires a listener to take into account the position of both the target and the background noise, and in some cases requires the listener to turn away from the target. Listeners in the real world, on the other hand, face an acoustic environment that rarely consists of a single target sound and a single, localizable distractor sound in a sound-attenuating room. A strategy of maximizing target level may have the advantage of being simple to do in a real and noisy environment where the spatial position of multiple noise sources may be difficult or impossible to determine. It may, however, be worthwhile to inform listeners of decidedly non-ideal orientations and ways of using head movements to improve SNR in the real world.
